# Beyond red/far‐red sensing: phytochrome perception of the marine light field by microalgae

**DOI:** 10.1111/nph.71128

**Published:** 2026-03-26

**Authors:** Carole Duchêne, Marianne Jaubert, Angela Falciatore

**Affiliations:** ^1^ Department of Algal Development and Evolution Max Planck Institute for Biology Tübingen 72076 Tübingen Germany; ^2^ Laboratoire de Photobiologie et Physiologie des Plastes et des Microalgues, UMR7141, CNRS, Sorbonne Université, Institut de Biologie Physico‐Chimique, F‐75005 Paris France

**Keywords:** diatom, marine environment, microalga, photoperception, phytochrome, underwater light field

## Abstract

Phytochromes (PHYs) are a major group of photoreceptors, described as red and far‐red light sensors in land plants. Recent genomic and metagenomic explorations have revealed the presence of PHYs also in various eukaryotic microalgae originating from distinct endosymbiotic events. Growing evidence indicates that these PHYs are spectrally and functionally tuned to shorter wavelengths, which are prevalent in the aquatic environments as depth increases. Investigations using emerging phytoplankton model species, along with environmental surveys, are uncovering new PHY‐mediated responses that likely influence their growth and distribution in marine environments. This Tansley Insight explores the implications of these discoveries for understanding the evolution and functional significance of this major photoreceptor class in the upper ocean, where light drives both energy and information flow.

**Table:** 

	Contents	
	Summary	2837
I.	Introduction	2837
II.	Microalgal PHY distribution through genomic and metagenomic lenses	2838
III.	Adaptation of PHY photoperception to aquatic environments	2839
IV.	Physiological role of PHY‐mediated underwater light sensing	2840
V.	Conclusions	2842
	Acknowledgements	2843
	References	2843

## I. Introduction

**Fig. 1:**
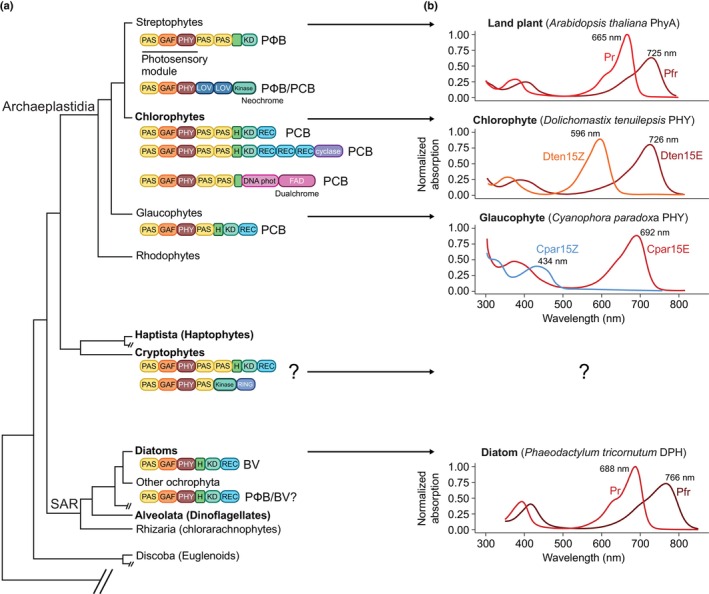
Diversity of phytochromes (PHY) in photosynthetic organisms. (a) Schematic phylogenetic tree of Eukaryotes (according to Williamson *et al*., 2025) highlighting the lineages containing photosynthetic organisms. Bold label depicts the top 5 most abundant algae in *Tara* Oceans metagenomic data (Pierella Karlusich *et al*., 2020). The PHY structures found in different aquatic photosynthetic organisms (Rockwell *et al*., 2014; Fortunato *et al*., 2016; Rockwell & Lagarias, 2020) are shown next to each lineage. DNA phot, DNA photolyase homologous domain; FAD, flavin adenine dinucleotide‐binding domain; GAF, cGMP phosphodiesterase/adenylate cyclase/FhlA; PAS, Per/ARNT/Sim; (H)KD, histidine‐kinase domain; Kinase, eukaryotic protein kinase; REC, response regulator receiver; RING, really interesting new gene; cyclase, adenylate/guanylate cyclase. Chromophores are indicated next to the structures; BV, biliverdin; PCB, phycocyanobilin; PФB, phytochromobilin. (b) Absorption spectra of representative PHY from each major photosynthetic lineage, with the wavelength of the main absorption peak for each form.

**Fig. 2:**
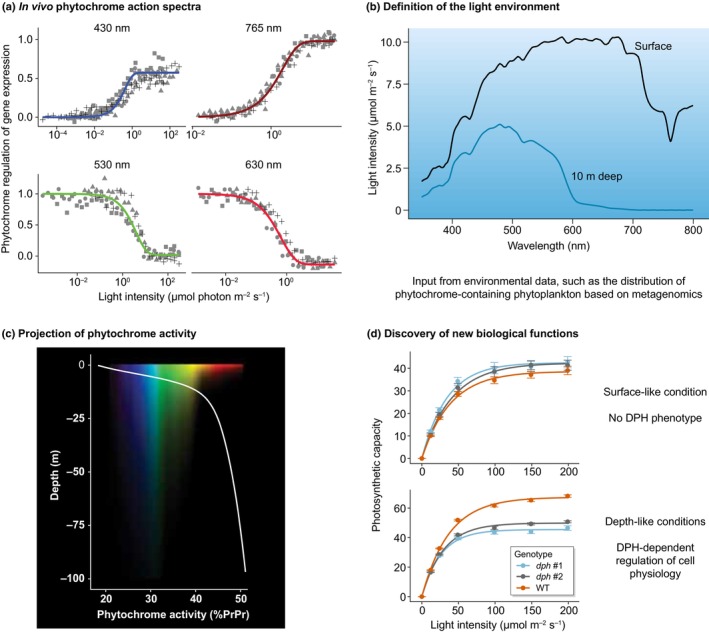
Power of combining phytochromes (PHY) action spectra with environmental light fields to discover new biological functions, as exemplified by the diatom phytochrome (DPH). (a) PHY action spectra are determined *in vivo* under controlled laboratory conditions. Wavelength and fluence‐dependence analyses of PHY‐mediated responses (gene expression changes in this example), along with information on PHY absorption spectra, can be used to parameterize models of PHY activity under a given light field. (b) Defining the environmental niche of PHY‐containing algae, for example, through metagenomics, helps to identify the environmental light spectra these algae may encounter. (c) Integrating this information with the PHY activity models established in ‘a’ allows for projecting PHY activity in relevant environments and formulating hypotheses about the environmental signals to which PHY responds. In the case of DPH, its activity reflects optical depth, leading to the hypothesis that DPH could help mediate acclimation to different light conditions that diatoms may encounter along the water column. (d) These hypotheses help to design key experiments in the laboratory to discover new photoreceptor's functions. In the example of DPH, analysis of the diatom wild‐type and a *dph* knockout acclimated under light conditions mimicking surface or depth revealed DPH‐dependent regulation of photosynthesis only under depth‐like conditions, thereby uncovering the first physiological function of PHY in marine algae. Given the strong effect of rapid light changes on the photosynthetic process itself, it is important to note that photoreversibility, a hallmark of PHY activity, has not yet been demonstrated for DPH‐regulated photosynthesis. However, it has been demonstrated on other processes such as gene expression and collective behavior.

Phytoplankton include diverse photosynthetic microorganisms that make up less than 1% of Earth's total photosynthetic biomass. Yet, they are responsible for *c*. 50% of global primary productivity and oxygen production (Field *et al*., 1998). The vertical gradients of light and nutrients strongly influence primary productivity and the distribution of phototrophs throughout the photic zone (Box 1). The discovery of both conserved and novel, lineage‐specific photoreceptors in the microalgal genomes (e.g. Takahashi *et al*., 2007; Coesel *et al*., 2009; Djouani‐Tahri *et al*., 2011; Fu *et al*., 2014; Rockwell *et al*., 2014; Fortunato *et al*., 2016; Makita *et al*., 2021) provides growing evidence that these organisms use light not only as energy source but also to gather information about their environment, much like terrestrial plants. In addition to various blue light sensors belonging to different families (Coesel *et al*., 2021), microalgae also possess ‘canonical’ phytochromes (PHY), which have an N‐terminal photosensory core module responsible for light detection and a C‐terminal signaling output module (Fig. 1a; Box 2). Phytochromes use linear tetrapyrroles as chromophores and function as molecular photoswitches, shifting between active and inactive states depending on the available light wavelengths and intensities (see Rockwell & Lagarias, 2020). The central role of red/far‐red sensing by PHY in regulating terrestrial plant growth and development is well established (Mancinelli, 1994; Legris *et al*., 2019). PHYs also show minor absorption of blue light and in plants contribute to some blue light responses, although often not well disentangled from the action of blue light photoreceptors (Liu *et al*., 2011; Wang *et al*., 2018; Belmonte *et al*., 2025). Conversely, it has remained unclear until recently whether the underwater light field can provide signals detectable by algal PHY and whether these signals are ecologically significant for these organisms. Focusing on the PHY of eukaryotic microalgae (Fig. 1), this review summarizes recent efforts aimed at addressing this important question, which involves overcoming several challenges: (1) defining the ‘light environment’ experienced by algae containing PHYs, including the spectra and spectral variations they are exposed to; (2) characterizing the spectral properties and sensitivity of algal PHYs that mediate effective responses; and (3) integrating this information to design environmentally relevant experiments that probe PHY function in algae physiology (Fig. 2).

Box 1 **The vertical dimension of the underwater water column structures the phytoplankton life.**

Phytoplankton growth and distribution depend on the availability of nutrients and light, which have opposite gradients along the water column. Temperature (a) varies with depth as well, but in the upper layers, it changes more gradually than the daily fluctuations observed on land (Mann & Lazier, 2006). In low‐turbulence waters, surface nutrients – such as nitrates, nitrites, phosphates, and silicates – are depleted by phytoplankton and transported downward through sinking dead cells, fecal pellets, and aggregates (b). Light intensity decreases exponentially with depth due to water absorption, which also modifies its spectrum (c). Long and short wavelengths are absorbed preferentially, enriching the underwater light field in blue and green light. Phytoplankton pigments, mainly chlorophylls, absorb blue and red, whereas dissolved organic matter and nonalgal particles primarily affect short wavelengths through absorption or scattering. As a result, underwater light fields are enriched in blue light in clear ocean waters, whereas coastal waters with organic matter and sediments are shifted toward green. Models and data can predict marine light fields (Morel & Maritorena, 2001). The upper part of the water column where light enables photosynthesis is called the (eu)photic zone, usually defined as the layer where light exceeds 1% of surface irradiance (100–200 m). Recent estimates based on nonphotosynthetic light‐controlled responses extend the limits of the photic zone (Davies & Smyth, 2025). In this strongly depth‐varying light field, physical mixing plays a crucial role. Thermal stratification can limit mixing to the upper meters, while seasonal cooling (e.g. winter in temperate regions) can cause intense mixing often well below 100 m, pushing phytoplankton below the photic zone and reducing primary production. The depth dimension thus strongly influences phytoplankton life. Underwater light perception by phytochrome may serve several, as yet unexplored roles, including responses to other abiotic cues that vary with depth and even the ability to detect neighboring organisms in high‐density, seasonal algal blooms.

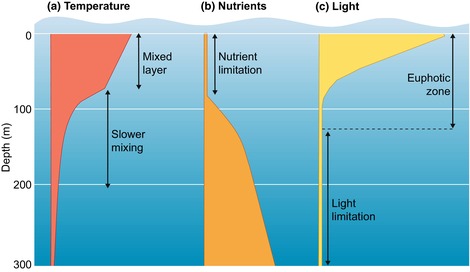

Box 2 **Phytochrome photobiology glossary.**

**Phytochrome (PHY) photoreceptor:** Made of an apoprotein on which a chromophore is bound for light perception.

**Photosensory module:** N‐terminal part of the protein where the chromophore is bound, responsible for light perception. Usually, it is sufficient to recapitulate the light absorption features of the full‐length PHY. It consists of three consecutive domains: PAS, GAF, and PHY that adopt a conserved knotted structure, highly conserved among all the canonical PHY.

**Output domain:** Portion of the protein initiating signaling cascade. PHY exhibit diversification of their output modules across different clades.

**PHY chromophores:** Linear tetrapyrroles deriving from heme, usually bound covalently to a cysteine residue either in the GAF domain or in the N‐ter PAS domain. The nature of tetrapyrrole is largely clade‐specific and influences the absorption spectra of the holoprotein: phytochromobilin is the chromophore of plant PHY; phycocyanobilin is the chromophore of cyanobacteria, streptophyte algae and the spectrally tuned chlorophyte and glaucophyte PHY; biliverdin, the chromophore bound by bacterial, fungi, and diatom PHY, generates the most red‐shifted PHY absorption peaks (both in the so‐called Soret band in the blue region and the red and far‐red ones).

**Photoreversibility:** Property of PHY to switch from one form to another when exposed to specific wavelengths of light and revert back to the original form upon exposure to different light. Responses triggered by activating light can be reversed by subsequent exposure to inactivating light.

**Action spectra:** Effectiveness of various wavelengths on a biological process. The shape of an action spectrum reveals information about the pigment‐binding protein involved in the regulation of the process.

## II. Microalgal PHY distribution through genomic and metagenomic lenses

Around the same time as the discovery of PHYs in land plants, action spectra of chloroplast movement in *Mougeotia* (Haupt, 1973) demonstrated red/far‐red light perception in macroalgae. However, it was only with the advent of the genomic era that PHY diversity started to be revealed in eukaryotic microalgae. Canonical PHYs have been identified in the genomes of algae derived from primary endosymbiosis events, such as chlorophytes, the algal lineage sister to streptophytes in Archaeplastida, and glaucophytes – along with organisms that originated through secondary endosymbiosis, including cryptophytes and ochrophytes, the photosynthetic stramenopiles with diatoms and multicellular brown algae (Fig. 1a; Box 2; Rockwell & Lagarias, 2020). The latter possess PHY phylogenetically related to that found in nonphototrophs, such as fungi (Duanmu *et al*., 2014; Fortunato *et al*., 2016). However, PHY is not evenly distributed among algae. It has been detected in some, but not all, green algae (Duanmu *et al*., 2014) and found in oceanic diatoms only within the Thalassiosirales and Cymatosirales (Duchêne *et al*., 2025). Multiple PHY genes are present in the genomes of certain benthic diatoms and brown macroalgae. The presence of PHY in some macroalgal viruses further suggests acquisition via viral infection (Fortunato *et al*., 2016). Therefore, these distribution patterns suggest a complex, still unresolved evolution of PHY in microalgae, likely involving multiple acquisition events and lineage‐specific gene loss and duplication. Recent studies discovered a new photoreceptor, called dualchrome, featuring the fusion of a full PHY with light‐sensing domains characteristic of a cryptochrome blue light photoreceptor. Found in the green alga *Pycnococcus provasolii* and metagenome‐assembled genomes (Makita *et al*., 2021; Delmont *et al*., 2022), dualchrome is reminescent of the Neochrome, a fusion of PHY and phototropin in ferns, hornwort, and streptophyte algae (Suetsugu *et al*., 2005; Li *et al*., 2014). The extensive ‐omic datasets generated by large‐scale oceanographic campaigns, including *Tara* Oceans (Tara Oceans *et al*., 2022), now make it possible not only to broaden our analyses of photoreceptors in aquatic microbes – thereby refining our understanding of their evolution – but also to examine their distribution throughout the global ocean and assess how photoreceptor abundance correlates with environmental parameters. This is well exemplified by the study of diatom phytochromes (DPHs), which reveal a strong biogeographic pattern: PHY‐bearing diatoms are predominantly found in temperate and polar regions and are undetected in tropical areas, whereas blue‐light photoreceptors such as Aureochromes are found everywhere (Duchêne *et al*., 2025). The high‐latitude distribution correlates with parameters characteristic of temperate and polar regions, including low temperatures, higher phytoplankton concentrations, and deeper water mixing, as well as their seasonal variations. These environmental data may provide clues to understanding PHYs' informational role in the ocean, thereby enabling the formulation of new hypotheses regarding the ecological advantages conferred by light sensing in specific environments. As elaborated in the subsequent paragraphs, this requires, however, characterization of the photoreceptor's light‐sensing abilities, which cannot be inferred only from protein sequence data.

## III. Adaptation of PHY photoperception to aquatic environments

Initial information on microalgae PHY photosensing can be obtained by analyzing their absorption spectra on *in vitro* recombinant holophytochromes (Wahleithner *et al*., 1991). This approach revealed that some algal PHYs display variations in their absorption spectra, proposed to be an adaptation to the aquatic light environment in a process called spectral tuning (Rockwell *et al*., 2014). Indeed, PHYs of prasinophytes (chlorophytes) absorb at shorter wavelengths than their land plant counterparts, down to the yellow band (Fig. 1b). Those from glaucophytes exhibit even more shifted forms, with one form of the PHY completely losing the ability to sense red or far‐red light (Rockwell *et al*., 2014; Fig. 1b). Interestingly, the PHY part of the dualchrome in Prasinophytes also exhibits a tuned orange/far‐red absorption form, combined with blue light sensing of the cryptochrome part in a single protein (Makita *et al*., 2021). An apparent opposite and counterintuitive situation was found for all the DPH cloned to date from diverse diatom species and environmental DNA, which do not show any signs of spectral tuning or structural variation (Fig. 1; Box 2). Initially shown to trigger gene expression changes under far‐red light (Fortunato *et al*., 2016), an in‐depth exploration of the DPH action spectra (Duchêne *et al*., 2025) revealed strong activation of DPH‐dependent responses also in blue light, whereas inhibition was effective in red and green. Blue light sensing may result from DPH binding biliverdin as a chromophore, with the absorption peaks (the major in red or far‐red and the minor in blue) shifted toward longer wavelengths compared to plant PHY (Box 1). Diatom phytochromes therefore appear as a functional photoreversible sensor along the entire light spectrum, potentially able to detect the red : far‐red ratio, as do plant PHYs, but also the blue : red and even the blue : green ratio. Considering that in the marine environment, far‐red light is present only at the surface, underwater DPH is *de facto* a sensor of blue‐, green‐, and red‐light ratios (Fig. 2b,c).

Furthermore, the modeling of the DPH activity in the field, based on *in vivo* action spectra measured in the laboratory and environmental light spectra measured along the water column, showed that DPH activity increases with depth (Fig. 2c). This behavior reflects a dynamic balance between activating wavelengths, present at depth (e.g. blue light), and inhibitory wavelengths, which dominate near the surface (mostly red light). Together with data on the environmental distribution of DPH‐containing diatoms, these observations have guided the first functional investigation of PHY in these prominent phytoplankton. PHY function remains cryptic in all other algal lineages.

## IV. Physiological role of PHY‐mediated underwater light sensing

The high sensitivity of DPH throughout the entire water column has inspired new hypotheses about its role as a depth sensor. So far, optical depth detection has been primarily associated with the activity of the photosynthetic apparatus, potentially triggering different retrograde pathways (e.g. changes in the redox state of plastoquinone pool) at various depths (Behrenfeld *et al*., 2016). A comparative photophysiology analysis mimicking the underwater light field in wild‐type and *dph* knockout mutants of *Thalassiosira pseudonana*, a representative planktonic diatom, indeed revealed a new role for DPH in controlling photosynthesis (Fig. 2d). This function was observed only when diatoms acclimated to low‐ and blue‐light conditions typical of deep water were exposed to higher light intensities. Conversely, DPH seems to have little impact on diatoms acclimated to higher light intensities and broader spectral ranges near the surface, possibly because other plastid‐driven mechanisms or additional photoreceptors compensate for or bypass the loss of DPH (Duchêne *et al*., 2025). This discovery also offers a plausible explanation for the different distribution of diatoms with DPH in temperate and polar regions. In these environments, DPH may provide an adaptive advantage by helping these microalgae anticipate and adapt to sudden changes in light intensity caused by water‐column mixing and rapid cellular vertical displacement. By contrast, in equatorial regions – where water is more stratified – organisms are less likely to encounter such dynamic light fluctuations, making DPH‐mediated photosynthetic control less relevant. However, latitudinal differences are also associated with changes in photoperiod and temperature. Interestingly, the latitudinal distribution of DPH mirrors that of a core circadian clock variant potentially involved in photoperiod adaptation in cosmopolitan Mamiellophyceae (Chlorophytes) species that lack a functional circadian system at low latitudes (Rigonato *et al*., 2025). In plants, PHYB integrates light and temperature signals through the temperature dependence of its spontaneous reversion to the dark state (Jung *et al*., 2016; Legris *et al*., 2016). Further investigation of the effect of temperature on the dark relaxation and photoequilibrium of aquatic PHYs will clarify whether these receptors share similar properties, which could be exploited for day length and temperature perception at high latitudes.

The role of DPH extends beyond regulating photophysiology. Recent studies using laser diffractometry show that some diatom species use light signals to collectively change their orientation and sinking rates, which could be important in marine ecology (e.g. for finding a partner for sexual reproduction in low‐mixing environments at depth). Analysis of *Phaeodactylum tricornutum* wild‐type and *dph* mutants indicates that DPH is necessary to establish coordinated social behavior (Font‐Muñoz *et al*., 2026). Similar to the DPH‐driven changes in gene expression, synchronized cell movements are triggered by blue or far‐red light and turned off by red, and proportional to the blue/red or far‐red/red ratio. Evidence also indicates that this collective behavior involves the emission and detection of natural fluorescence signals (Font‐Muñoz *et al*., 2021), which can modify the underwater light field (Font‐Muñoz *et al*., 2025). The ability of PHYs to detect red light originating from Chl fluorescence has been previously proposed (Ragni & d'Alcalà, 2004; Fortunato *et al*., 2016; Font‐Muñoz *et al*., 2021). However, for such a mechanism to function, PHY must discriminate between external light sources, fluorescence of its own cell and those emitted by neighbors – a distinction that can be extremely challenging except under conditions such as dense algal blooms or when cells form aggregates. This hypothesis, to be tested, would require accurate measurement of internal red‐light sources and determination of DPH intracellular location, which might change under different conditions. Other biotic cues could also influence DPH‐mediated responses, as demonstrated in some bacteria where quorum‐sensing and light‐sensing systems work together to control biofilm formation (Mukherjee *et al*., 2019).

## V. Conclusions

Consistent with earlier findings for bacterial rhodopsins (Man *et al*., 2003), the spectral and functional tuning of microalgae PHYs reflects the adaptation of aquatic photoreceptors to the abundant, and likely more informative, shorter wavelengths underwater. The detection of blue and green light by DPH, initially overlooked, emerged only when assessing its *in vivo* action spectra. The high sensitivity of this PHY to blue light, coupled with the underwater enrichment of this light color, makes this receptor an exquisite detector at depth, even when light intensities are too low to trigger responses via photosynthetic activity.

The spectrally tuned algal PHY from Archaeplastida (Fig. 1) likely evolved independently of the bacterial‐like PHY found in diatoms (Duanmu *et al*., 2014). Therefore, short‐wavelength sensing may have arisen independently in different aquatic algal lineages. By contrast, red and far‐red light sensing may have been favored in terrestrial plants because these wavelengths are abundant, and their ratio changes significantly in specific environments, such as under forest canopies or during sunrise.

The study of gene‐expression and collective‐behavior changes mediated by DPH also emphasizes that algal PHYs utilize band‐ratio information, as terrestrial PHYs do. This function could be used by phytoplankton to adjust growth and life strategies within the photic zone, where the underwater light field changes across space and time. Recent observations suggest that the exploitation of changes in underwater wavelengths may also have been pursued by other algae, although with different solutions. Organisms lacking PHY might accomplish these functions through the expansion of the absorption spectrum of blue light photoreceptors (e.g. the Chlamydomonas aCry absorbing also in red (Beel *et al*., 2012)) or combined action of multiple receptors, as proposed for the LOV‐histidine kinase and rhodopsin histidine kinase in the green alga *Ostreococcus* (Thommen *et al*., 2015).

The conservation of signaling pathways downstream of PHY activation is another interesting open question: if green algae and land plant PHY share some signaling mechanisms (Duanmu *et al*., 2014), nothing is known about diatom ones. It will be interesting to determine whether DPH exhibits different cellular localization throughout the diel cycle, as observed in Micromonas, since this could affect diel and photoperiodic responses.

Most of the data presented in this review come from a limited number of algal PHYs from three lineages: glaucophyte, chlorophyte, and ochrophyte. Cryptophyte PHYs are evolutionarily close to those of Archaeplastida (Rockwell & Lagarias, 2020), but nothing is yet known about their light‐sensing abilities. The growing amount of genomic and metagenomic data offers unprecedented opportunities to expand research on PHYs evolution across organisms from different lineages and their environmental distribution, which has so far been investigated primarily for DPH. Along with the development of novel genetically tractable model algae and quantitative analysis of wavelength‐ and intensity‐dependent reactions *in vivo*, this will support further research on PHY functional diversification.

Integrating the light‐sensing properties of PHY with the light field in which the organism thrives is a powerful approach to designing experimental conditions to test for new PHY‐regulated phenotypes. We illustrate here how this information has guided investigations in diatoms (Fig. 2), but this approach can be expanded to other phytoplankton or aquatic PHY‐containing organisms (bacteria, fungi, and even viruses).

Incorporating data on photoreceptor‐controlled processes into ecological models will also enable more precise estimates of phytoplankton activity across diverse environments. This is especially important as climate change continues to alter the temporal and geographic distribution of these organisms, changing the environmental conditions they encounter throughout the water column, at different times of day, and in different seasons.

## Competing interests

None declared.

## Author contributions

CD, MJ and AF contributed equally to the conceptualization of the review and the writing of the main text. CD generated the figures.

## Disclaimer

The New Phytologist Foundation remains neutral with regard to jurisdictional claims in maps and in any institutional affiliations.
